# Single-ion magnetism in the extended solid-state: insights from X-ray absorption and emission spectroscopy†

**DOI:** 10.1039/d0sc03787g

**Published:** 2020-10-07

**Authors:** Myron S. Huzan, Manuel Fix, Matteo Aramini, Peter Bencok, J. Frederick W. Mosselmans, Shusaku Hayama, Franziska A. Breitner, Leland B. Gee, Charles J. Titus, Marie-Anne Arrio, Anton Jesche, Michael L. Baker

**Affiliations:** The University of Manchester at Harwell, Diamond Light Source Harwell Campus OX11 0DE UK michael.baker@manchester.ac.uk; Department of Chemistry, The University of Manchester Manchester M13 9PL UK; EP VI, Center for Electronic Correlations and Magnetism, Institute of Physics, University of Augsburg D-86159 Augsburg Germany; Diamond Light Source, Harwell Science and Innovation Campus Chilton Didcot OX11 0DE UK; Department of Chemistry, Stanford University Stanford CA 94305 USA; Department of Physics, Stanford University Stanford CA 94305 USA; Institut de Minéralogie, de Physique des Matériaux et de Cosmochimie, CNRS, Sorbonne Université, IRD, MNHN, UMR7590 75252 Paris Cedex 05 France

## Abstract

Large single-ion magnetic anisotropy is observed in lithium nitride doped with iron. The iron sites are two-coordinate, putting iron doped lithium nitride amongst a growing number of two coordinate transition metal single-ion magnets (SIMs). Uniquely, the relaxation times to magnetisation reversal are over two orders of magnitude longer in iron doped lithium nitride than other 3d-metal SIMs, and comparable with high-performance lanthanide-based SIMs. To understand the origin of these enhanced magnetic properties a detailed characterisation of electronic structure is presented. Access to dopant electronic structure calls for atomic specific techniques, hence a combination of detailed single-crystal X-ray absorption and emission spectroscopies are applied. Together K-edge, L_2,3_-edge and Kβ X-ray spectroscopies probe local geometry and electronic structure, identifying iron doped lithium nitride to be a prototype, solid-state SIM, clean of stoichiometric vacancies where Fe lattice sites are geometrically equivalent. Extended X-ray absorption fine structure and angular dependent single-crystal X-ray absorption near edge spectroscopy measurements determine Fe^I^ dopant ions to be linearly coordinated, occupying a *D*_6h_ symmetry pocket. The dopant engages in strong 3dπ-bonding, resulting in an exceptionally short Fe–N bond length (1.873(7) Å) and rigorous linearity. It is proposed that this structure protects dopant sites from Renner–Teller vibronic coupling and pseudo Jahn–Teller distortions, enhancing magnetic properties with respect to molecular-based linear complexes. The Fe ligand field is quantified by L_2,3_-edge XAS from which the energy reduction of 3d_*z*^2^_ due to strong 4s mixing is deduced. Quantification of magnetic anisotropy barriers in low concentration dopant sites is inhibited by many established methods, including far-infrared and neutron scattering. We deduce variable temperature L_3_-edge XAS can be applied to quantify the *J* = 7/2 magnetic anisotropy barrier, 34.80 meV (∼280 cm^−1^), that corresponds with Orbach relaxation *via* the first excited, *M*_J_ = ±5/2 doublet. The results demonstrate that dopant sites within solid-state host lattices could offer a viable alternative to rare-earth bulk magnets and high-performance SIMs, where the host matrix can be tailored to impose high symmetry and control lattice induced relaxation effects.

## Abstract 2

Taking advantage of synchrotron light source methods, we present the geometric and electronic structure of iron doped in lithium nitride.

## Graphical abstract

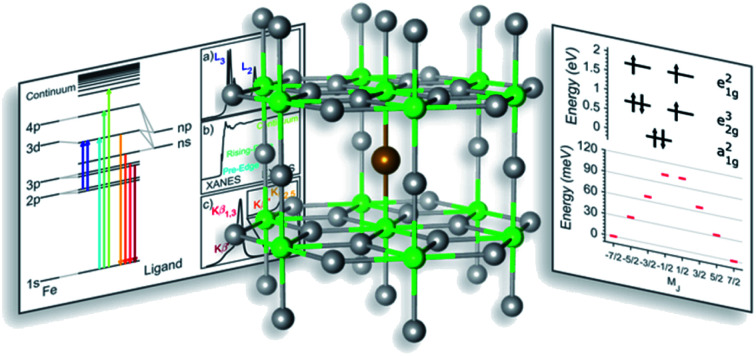

## 1 Introduction

Linearly coordinated transition metal ions can exhibit first-order spin–orbit coupling which gives rise to large magnetic anisotropy barriers and bi-stability of magnetisation. An example of this is Fe doped in lithium nitride (Li_2_(Li_1−*x*_Fe_*x*_)N). The magnetic anisotropy energy of Li_2_(Li_1−*x*_Fe_*x*_)N exhibits an observed coercivity field of more than 11 T, exceeding even the largest values observed in rare-earth-based permanent magnets.^1^ Consequently, the underlying electronic structure of Li_2_(Li_1−*x*_Fe_*x*_)N is of relevance to the search for alternatives to rare-earth materials. Furthermore, since large single-crystals can be prepared,^2^ and the concentration of Fe sites (*x*) can be controlled, Li_2_(Li_1−*x*_Fe_*x*_)N prepared at low doping concentrations is proposed as single-ion like and therefore a solid-state equivalent^3^ to molecular based single ion magnets (SIMs). SIMs are complexes which display slow magnetic relaxation, and magnetic remanence relevant to nano-scale information storage technologies. Notable recent examples of linearly coordinated transition metal SIMs include a Co^II^(C(SiMe_2_ONaph)_3_)_2_ (where naph is a naphthyl group) complex with a non-aufbau (d_*x*^2^−*y*^2^_,d_*xy*_)^3^(d_*xz*_,d_*yz*_)^3^(d_*z*^2^_)^1^ configuration and resultant *L* = 3 ground-state orbital angular momentum.^4^ Another intriguing result are unusual 3d ligand fields. A *D*_∞h_ crystal field transforms the 3d-orbitals to a A_1_(3d_*z*^2^_) singlet at highest energy, followed by a E_1g_(3d_*xz*_,3d_*yz*_) doublet at an intermediate energy and a E_2g_(3d_*xy*_,3d_*x*^2^−*y*^2^_) doublet at lowest energy. However, multi-reference calculations based on the crystal structures of linear complexes, [M^I^(N(SiMe_3_)_2_)_2_]^−^ (where M = Cr, Mn, Fe, Co) all predict a d-orbital splitting with 3d_*z*^2^_ at lowest energy.^5^ Reasoning for this is due to strong 4s–3d_*z*^2^_ mixing that weakens the anti-bonding character of the metal ion 3d_*z*^2^_ orbital. Experimental evidence and the associated implications of 4s–3d_*z*^2^_ mixing on magnetic properties have been investigated on [Fe^I^(C(SiMe_3_)_3_)_2_]^−^.^6^ Calculations, based on the crystal structure, propose a a_1g_^2^e_2g_^3^e_1g_^2^ ground state configuration with an almost unquenched *L* = 2 orbital angular momentum. These calculations are supported and found consistent with Mössbauer spectroscopy data^7^ and high-resolution single-crystal crystallography provides the first experimental evidence of 3d_*z*^2^_^2^ electron occupation from electron density analysis.^8^ However, despite the increasing number of reports of new linear transition metal SIMs^9^ there have been very few experimental studies beyond the characterisation of orientation averaged magnetism. In this paper we demonstrate X-ray absorption spectroscopies as an accurate means to characterise the geometric and electronic structure of two coordinate transition metal SIMs.

**Fig. 1:**
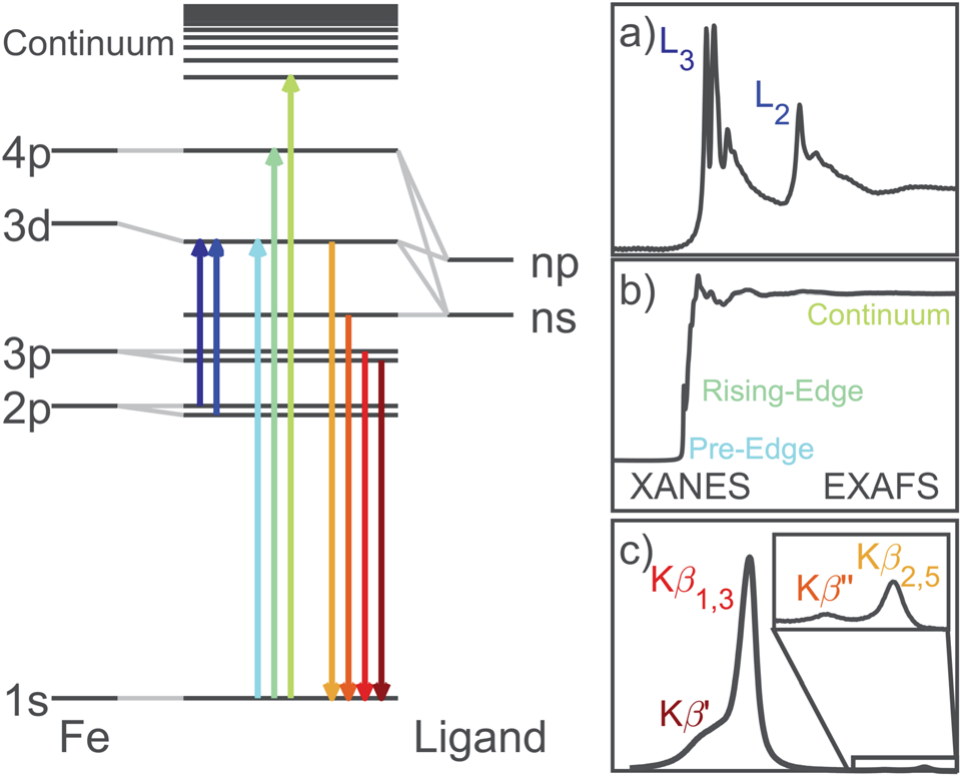
Illustration of the Fe X-ray spectroscopic techniques with the associated transitions. (a) L_2,3_-edge absorption probes unoccupied 3d orbitals. Spin–orbit coupling within the 2p^5^ core-hole splits the absorption into the 2p_1/2_ and 2p_3/2_ edges. (b) K-edge pre-edge, edge and EXAFS correspond to 1s absorptions into unoccupied 3d, 4p and continuum respectively. (c) Following the ejection of a 1s electron, Kβ XES involves the decay of Fe 3p and occupied valence electrons into the 1s core-hole.

We report results of extended X-ray absorption fine structure (EXAFS), X-ray absorption near edge spectroscopy (XANES), Kβ X-ray emission spectroscopy (XES) and L_2,3_-edge X-ray absorption spectroscopy (XAS) single-crystal measurements on Li_2_(Li_1−*x*_Fe_*x*_)N. Each of these techniques have specific sensitivities associated with transition selection rules and generated core-holes. A schematic overview of the spectroscopic techniques and associated transitions are shown in Fig. 1. K-edge XANES probes unoccupied 4p orbitals with 1s → 4p dipole transitions and unoccupied 3d orbitals *via* the much weaker intensity pre-edge, 1s → 3d, transitions. The technique has particular sensitivity to local coordination symmetry, making it ideally suited for probing distortions from a linear, *D*_∞h_, to a bent, *C*_2v_, coordination. Bending leads to a pseudo Jahn–Teller effect that mixes 4p_*x*,*y*_ character into 3d_*xz*,*yz*_. This mixing can be clearly identified since it drives strong dipole intensity enhancement in the pre-edge. EXAFS quantifies interference effects due to electron scattering from the surrounding atoms. Kβ XES involves the ionisation of a 1s electron and the detection of photons emitted from occupied 3p and occupied valence-electrons filling the 1s core-hole. Inter-shell 3d–3p Coulomb exchange makes Kβ XES a sensitive probe of 3d spin-state in Li_2_(Li_1−*x*_Fe_*x*_)N as a function of concentration, *x*.^10^ L_2,3_-edge XAS probes 2p → 3d dipole transitions providing direct experimental access to the ligand field, related 4s–3d_*z*^2^_ mixing, spin–orbit coupling and the resultant anisotropy barrier.^11^

**Fig. 2:**
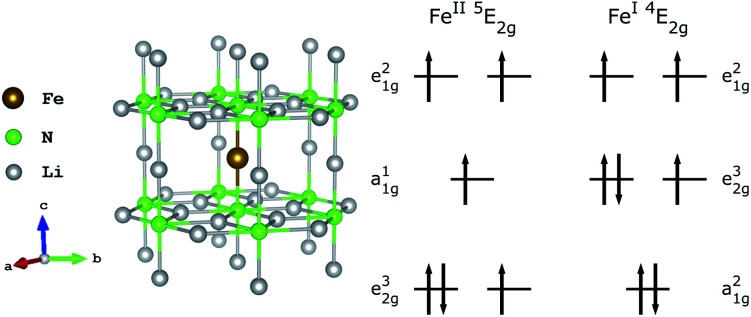
(left) Structure of Li_2_(Li_1−*x*_Fe_*x*_)N; Fe substitution within α-Li_3_N matrix at the 1*b* Wyckoff position. (right) Proposed Fe^II^ and Fe^I^ electron occupations.^20^

Magnetisation studies for low Fe doping concentrations show hysteresis is maintained up to 16 K with sweep rates of 15 mT s^−1^,^12^ which is the largest temperature reported for a transition metal SIM. The effective energy barrier to magnetisation reversal is estimated between 37.1 and 40.2 meV (298.9 and 324.6 cm^−1^).^12^ Below a blocking temperature of ∼10 K relaxation to magnetisation becomes temperature independent, with an exceptionally long magnetic relaxation time of *τ* > 10^4^ s.^13^ However, despite several theoretical^1,14,15^ and experimental^13,16–19,55^ studies, the electronic structure of Li_2_(Li_1−*x*_Fe_*x*_)N remains a matter of considerable contention. Even the oxidation state of Fe sites has been brought into question by recent *ab initio* calculations proposing the presence of Li-ion vacancies coupled to Fe^II^ sites.^20^Fig. 2 shows the proposed structure of a single Fe dopant site present within Li_2_(Li_1−*x*_Fe_*x*_)N and possible ground state electronic configurations for both the divalent and mono-valent situations. Additional open questions arise due to the inaccessibility of low concentration Fe sites embedded within the host structure, and presence or absences of Fe dopant clustering effects and concentration dependencies. In this paper we apply the range of X-ray spectroscopies that selectively characterise different aspects of electronic structure, from which we identify Li_2_(Li_1−*x*_Fe_*x*_)N is a high symmetry solid state SIM clear of stoichiometric vacancies where Fe lattice sites are geometrically equivalent. The geometric and electronic structure of Li_2_(Li_1−*x*_Fe_*x*_)N is compared against molecular based SIMs and important insights into the origin of high temperature magnetic blocking and exceptionally long magnetic relaxation times observed in Li_2_(Li_1−*x*_Fe_*x*_)N are obtained.

## 2 Experimental section

### 2.1 Preparation of Li_2_(Li_1−*x*_Fe_*x*_)N

Single crystal growth of Li_2_(Li_1−*x*_Fe_*x*_)N was achieved through a Li-rich solution to a desired concentration, *x*, and verified by inductively coupled plasma mass spectrometry (detailed information on crystal synthesis reported by Jesche and Canfield in ref. 2). Li_2_(Li_1−*x*_Fe_*x*_)N crystallises as a hexagonal lattice of Li_2_N layers alternating with Li_1−*x*_Fe_*x*_ planes perpendicular to the crystallographic *c* axis. Dopant Fe site substitution replaces a Li-1*b* Wyckoff position ion, Fig. 2. Crystal growth stipulates the crystallographic *c* axis (magnetic easy-axis) to be oriented surface normal enabling accurate mounting of single crystals to easily perform angular dependence measurements.

### 2.2 K-edge XANES, EXAFS and Kβ XES measurements

XANES, EXAFS and Kβ XES measurements were performed using the high-resolution fluorescence-detection available at the I20-scanning beam-line at Diamond Light Source, UK, and exploiting a four-bounce Si(111) monochromator for spectral purity.^21^ The XES measurements were collected by a medipix detector from three Si(531) analyser crystals. XANES and EXAFS measurements were detected with a 64 element Ge detector windowed to the Kα fluorescence line. XANES monochromatic energy ranges; 7000–7075 eV, 5 eV step size, 1 s integration (pre-edge), 7075–7100 eV, decreasing step size 5–0.5 eV, 1 s integration (rising-edge), 7100–7135.5 eV, 0.5 eV step size, 1 s integration (XANES), 7135.5–8100 eV, 0.04 Å, increasing integration time from 1–5 s (EXAFS). Kβ emission energy ranges; 7025–7080 eV, 0.3 eV step size, 1s int. time (Kβ mainline), 7080–7120 eV, 0.2 step size, 5 s integration time (valence-to-core). Several spectra were acquired for each doping concentration. Kβ measurements were performed with the incident energy set above the Fe K-edge at 8500 eV. The XANES, EXAFS and Kβ measurements were performed at room temperature. Additional Kβ measurements at 80 K exhibited negligible difference with respect to measurements at room temperatures. To minimise diffraction induced distortions within EXAFS spectra, crystals of Li_2_(Li_1−*x*_Fe_*x*_)N were ground into powdered pellets where possible. However, it is reported that the host crystal Li_3_N exhibits an additional high pressure phase, of which grinding can induce a partial phase transformation of the Li_3_N lattice from the α to β phase.^22^ The XANES spectra of α and β Li_2_(Li_1−*x*_Fe_*x*_)N differ significantly. Therefore XANES measurements were performed on both single-crystal and powder samples, from which it was deduced that only the lowest concentration Li_2_(Li_1−*x*_Fe_*x*_)N sample was affected by grinding. For this reason the measurements performed on Li_2_(Li_1−*x*_Fe_*x*_)N for *x* = 0.0020(5) were on a single crystal while for *x* = 0.0063(4) and 0.0093(6) were undertaken on powder samples. Powder samples were formed into pellets and mixed with boron nitride to an appropriate dilution to minimise self-absorption effects. Samples of Li_2_(Li_1−*x*_Fe_*x*_)N were prepared within an argon atmosphere glove-box (<0.5 ppm O_2_ and H_2_O) where single crystals and powders were encapsulated with Kapton tape. Measurements were performed within a nitrogen gas atmosphere. XANES and EXAFS analysis was undertaken within the Athena and Artemis packages.^23^ Background subtraction was undertaken with a linear fitting of the pre-edge and normalisation through a third order polynomial of the post-edge. Bond length (*R*) and Debye–Waller factor (*σ*) were used as variables of fitting for neighbouring lithium and nitrogen atoms. A Levenberg–Marquardt non-linear least-squares minimisation was applied for EXAFS fitting. Kβ XES spectra are normalised through a trapezoidal integration and the subtraction of a constant. Angular dependent XANES measurements were performed at the BL9-3 beamline at SSRL. The measurements were performed at 10 K in transmission mode on a 1 mm thick single crystal. These measurements were performed with monochromatic energy ranges; 6785–7085 6 eV step size, 1 s integration (pre-edge), 7085–7150 eV, 0.15 eV step size, 1 s integration (rising-edge), 7150–8359.5 eV, 0.5 eV step size, 1 s (post-edge region). Background subtraction of main K-edge XANES isolates the rising-edge peak from which Pearson VII peak fitting was undertaken through a least-squares minimisation, Fig. S3.†

### 2.3 L_2,3_-edge XAS measurements

L_2,3_-edge XAS measurements were performed at the I10 high field magnet end station at Diamond Light Source. Fast energy XAS scans were performed between 690–755 eV with 0.1 eV step sizes. The measurements were performed between 4.5–400 K within an ultra-high vacuum (10^−10^ bar). Detection was performed *via* total fluorescence yield in a back-scattering geometry using a 10 × 10 mm^2^ silicon diode with a 150 nm Al cover to filter out electrons. Single crystals of Li_2_(Li_1−*x*_Fe_*x*_)N are too insulating to obtain drain current detected XAS. Single crystals were mounted within an argon glovebox with Torr Seal epoxy resin and transferred to experimental chamber through a nitrogen purged glovebag. Background subtraction of the spectra was performed with a linear fitting of the pre-edge (690–700 eV) and normalisation through a linear fitting of the post edge (735–750 eV). The 2p_3/2_ and 2p_1/2_ continuum transitions were subtracted through a double arctangent function^24^ (further details see ESI Fig. S4†).

Ligand field multiplet simulations of the L_2,3_-edge XAS results were performed using the quantum many-body scripting language, Quanty.^25^ The Quanty input files for the simulation of L_2,3_-edge fluorescence XAS were adapted from templates generated in Crispy.^26^ Multiplet effects are described by the Slater–Condon–Shortley parameters, *F*^k^_pp_, *F*^k^_pd_ (Coulomb) and *G*^k^_pd_ (exchange), reduced to 80% of the Hartree–Fock calculated values to account for the over-estimation of electron–electron repulsion found for the free ion. The 2p^5^ spin–orbit coupling parameter *ξ*_2p_ is found consistent with the atomic value (8.202 eV). The 3d spin–orbit coupling parameters were obtained by fitting to the temperature dependence of the L_2,3_-edge XAS, giving *ξ*_3d_ = 0.052 and 0.068 eV for the initial and final states respectively. The presence of 4s mixing in linear transition metal complexes is known to weaken the 3d*σ* anti-bonding character and reducing the energy of the 3d_*z*^2^_ orbital. This effect is accounted for in a simple 3d ligand field model, where the relative energy of the orbitals are adjusted with parameters *D*_q_, *D*_t_ and *D*_s_, in the *D*_6h_ point group. The ligand field parameters describe the d-orbital degeneracy and energy splittings of the A_1g_(3d_*z*^2^_) singlet, and two E doublets, E_1g_(d_*xy*_,d_*yz*_) and E_2g_(d_*x*^2^−*y*^2^_,d_*xy*_). A local linear coordination geometry is characterised by a *D*_∞h_ ligand-field, and has equivalence with *D*_6h_ when *D*_q_ = 0. Broadening of the transitions as described by the core-hole lifetime was applied through a Lorentzian function over the L_3_ and L_2_ edge of 0.35 eV and 0.7 eV full width half maximum (FWHM) respectively. Gaussian broadening due to the instrumental resolution was set to 0.25 eV FWHM and simulated at 4.5 K.

### 2.4 Calculation details

The density functional theory (DFT) calculations presented in this work were performed using the plane-wave pseudopotential DFT method available within the codes Quantum-Espresso^27^ and CASTEP.^28^ Generalised-gradient approximation for the exchange-correlation energy was selected in the form of PBE functional.^29^ Ultrasoft pseudopotentials were used for PBE and PBE+U calculations, whereas relativistic ultrasoft pseudopotentials were used for the non-collinear calculations including spin–orbit coupling. The pseudopotentials for use with Quantum-Espresso were taken from the PSlibrary^30^ while the pseudopotentials for use with CASTEP were generated self consistently. A kinetic energy cutoff of 90 Ry for the wave function and of 900 Ry for the charge density together with a (6 × 6 × 6) Monkhorst–Pack *k*-point grid were determined as parameters for convergence calculations. A (10 × 10 × 10) *k*-point grid was instead used for the calculation of the density of states (DOS). Self-consistent calculations were performed to a convergence value of 1 × 10^−7^ eV. Due to the isolated nature of Fe atoms in Li_2_(Li_1−*x*_Fe_*x*_)N, we operated with a 3 × 3 × 3 supercell constructed from the hexagonal cell of Li_3_N having space group *P*6/*mmm*. The structure was relaxed so that the Fe–N and Fe–Li distances in the first coordination shells of iron matched the distances evaluated from the analysis of EXAFS results. The reliability of the experimentally evaluated structure for simulations was tested by completing a relaxation up to an energy change of 3 × 10^−6^ eV per atom, which produced a structure yielding a shorter Fe–N distance but a comparable density of states. A smearing of 0.01 Ry was applied to the computed eigenvalues in order to improve the *k*-point convergence. The angular dependence of Fe K-edge was calculated including the effects of core-hole^31^ and using the same *k*-point grid as previously used for the DOS. Ground state DFT was then expanded by expressing the exchange-correlation potential in terms of local-density band theory *via* the PBE+U method.^32^ The electronic properties were calculated with the simplified, rotational-invariant formulation developed within the linear response approach.^33^ An effective *U* value of 4 eV was included in such calculations, as previously estimated for similar compounds.^34^ Angular-momentum dependent orbital occupation was determined with Löwdin charge analysis on top of ground-state, converged DFT wavefunctions. X-ray absorption spectra were computed by extracting the matrix elements for electronic interband transitions from the ground state DFT including the local effects of 1s core-hole as implemented in the code CASTEP. Such calculations were accomplished in the aforementioned supercell, in order to avoid interactions between periodic images of the core excitation. An energy shift of 7110.5 eV was applied to match the experimental data and normalised through trapezoidal integration of simulated spectrum. Transition broadening as a consequence of instrumental resolution (Gaussian) and core-lifetime effects (Lorentzian) was set as 0.2 and 1.25 eV FWHM respectively.

## 3 Results and discussion

### 3.1 Extended X-ray absorption fine structure (EXAFS)

**Fig. 3:**
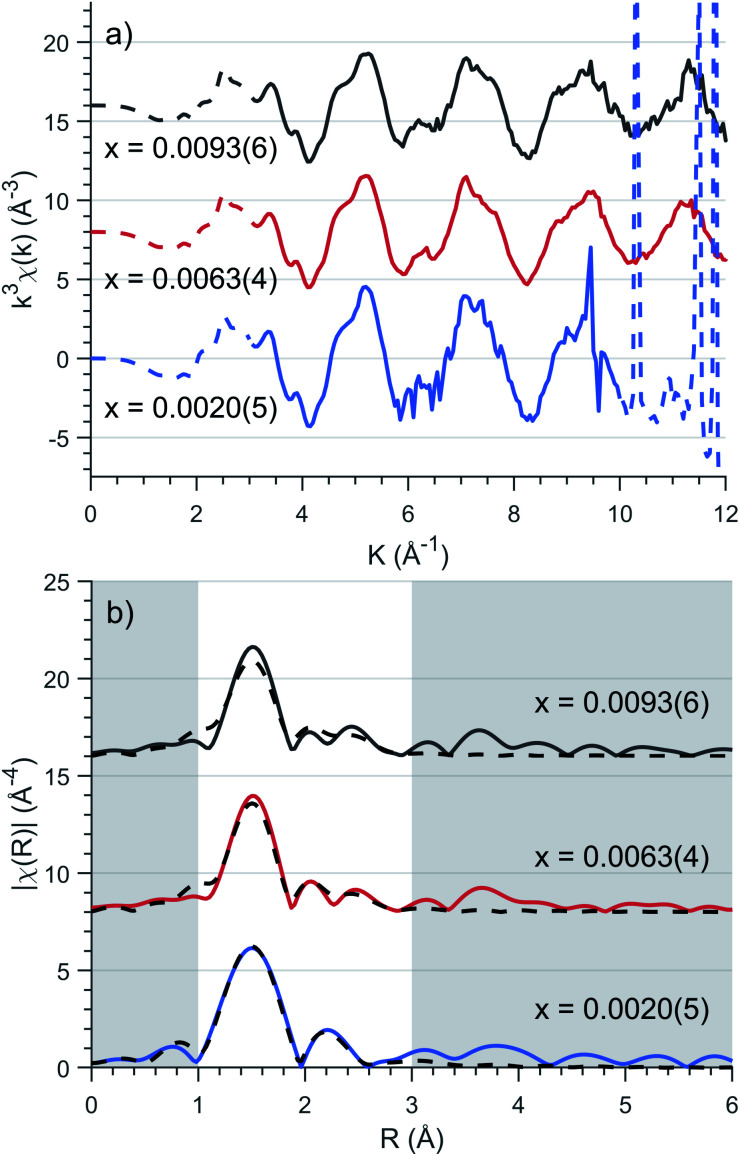
(a) Background subtracted *k*^3^ weighted XAFS spectra of various concentrations, *x*. Solid lines represent the Fourier transform region for EXAFS fitting. (b) Fitting of Fourier transformed *k*^3^-weighted EXAFS spectra for Li_2_(Li_1−*x*_Fe_*x*_)N as a function of concentration, *x*. EXAFS fitting is represented as dashed lines with corresponding parameters presented in Table 1.

To precisely quantify the local coordination environment at Fe sites EXAFS measurements were performed on samples with low dopant concentrations, where *x* = 0.0020(5), 0.0053(4) and 0.0093(6). The *k*^3^ weighted spectra are presented in Fig. 3a which highlight the EXAFS Fourier transform region as 3 ≤ *k* ≤ 10 Å^−1^ for *x* = 0.0020(5) and 3 ≤ *k* ≤ 12 Å^−1^ for *x* = 0.0053(4) and 0.0093(6) (*E*_0_ = 7113 eV). Single crystal measurements were performed with *E* 45° relative to the crystallographic *c* axis on the lowest concentration (*x* = 0.0020(5)) sample resulting in significant Bragg peaks for *k* values greater than 10 Å^−1^, requiring a reduced Fourier transform range.

EXAFS fitting was undertaken using a model including a single Fe atom dopant within α-Li_3_N as an initial structure (*a* = 3.652(8) Å and *c* = 3.870(10) Å, Fig. 2). The selected scattering paths were limited up to a radial distance of 3.5 Å; these included two single scattering pathways (Fe–N–Fe, Fe–Li–Fe) and one double scattering path (Fe–N–Li–Fe). Fitting of the experimental data was undertaken for each concentration individually. The EXAFS fit parameter results are presented in Table 1. The coordinated nitrogen atoms characterise the first spectral peak centred at 1.5 Å, Fig. 3b, while scattering from the hexagonally bonded lithium atoms in combination with the double scattering path characterise the remaining spectral features. The Fe–N bond lengths were determined to be 1.873(7) Å, 0.062(7) Å shorter than the equivalent Li–N bond length. This finding is consistent with X-ray diffraction results that show *c*-axis contraction and *a* and *b*-axis expansion on increasing Fe concentration.^12,16,35^ The length of the Fe–N bond is very short in comparison with values reported for two coordinate Fe^I^–N, including the linear complex [K(18-crown-6][Fe(N(SiMe_3_)_2_)_2_] (1.9213(6) Å)^36^ and the slightly bent [Fe(N(SiMe_3_)Dipp)_2_]^−^ (1.91 Å).^37^ The Fe–N length found for Li_2_(Li_1−*x*_Fe_*x*_)N compares more favourably with the linear divalent complex [Fe(N(SiMe_3_)Dipp)_2_] with a reported Fe^II^–N length of 1.853(1) Å.^38^ Many-body quantum chemistry calculations performed on Fe doped lithium nitride units [Fe^I^N_2_Li_14_]^9+^ and [Fe^II^N_2_Li_13_]^9+^ report bond lengths of 1.92 Å and 1.88 Å respectively.^20^ In summary, comparison with reported Fe–N bond lengths for two coordinate complexes might suggest a divalent oxidation state for Li_2_(Li_1−*x*_Fe_*x*_)N. Indeed this was the conclusion reported by Muller-Bouvet *et al.* (ref. 39) for Co doped lithium nitride, where EXAFS analysis included the pairing of dopants with Li vacancies at 2*c* sites, from which a formula of Li_3−2*x*_Co_*x*_N with a divalent Co oxidation state is proposed.^39^

**Table 1:** EXAFS fitting parameters of various Fe doping concentrations, *x* of Li_2_(Li_1−*x*_Fe_*x*_)N; *R*-factor (*R*), Energy alignment (Δ*E*), Fe–N and Fe–Li Bond Lengths (*R*_N_ and *R*_Li_ respectively) and Debye–Waller factor (*σ*^2^) for lithium (Li) and nitrogen (N) scattering atoms. Amplitude reduction factor, *S*_0_^2^ fixed to 0.92

*x*	0.0020(5)	0.0063(4)	0.0093(6)
*R*	0.004	0.028	0.061
Δ*E* (eV)	10.85 ± 0.93	9.35 ± 1.84	9.73 ± 2.85
*R*_N_ (Å)	1.873 ± 0.007	1.869 ± 0.015	1.868 ± 0.023
*σ*_N_^2^ (Å^2^) × 10^−3^	0.7 ± 0.5	3.4 ± 1.0	4.9 ± 1.6
*R*_Li_ (Å)	2.867 ± 0.015	2.840 ± 0.025	2.845 ± 0.039
*σ*_Li_^2^ (Å^2^) × 10^−3^	20.4 ± 3.0	19.6 ± 4.5	17.9 ± 5.8

The EXAFS measurements are consistent with isolated Fe dopants with no indication of clustering evidenced through the lack of strong features beyond the first structural peak at 1.5 Å. While not conclusive there is an observed increase in *R*-factor with concentration which could be attributed to the requirement of incorporating small contributions from Fe–Fe and Fe–N–Fe scattering paths within the *ab* plane and along the *c* axis respectively.

According to combinatorial analysis, the probability of locating *n* Li ions at the 8 possible neighbouring sites (6 perpendicular and 2 parallel to the crystallographic *c* axis) for a dopant Fe ion is expressed as: *W*_*n*_ = 8![*n*!(8 − *n*)!]^−1^(1 − *x*)^*n*^*x*^8−*n*^.^40^ At the highest doped concentration, *x* = 0.0093(6), the probability of all 8 neighbouring atoms being lithium is 92.8%, at which point there begins to be a non-negligible requirement of additional scattering pathways to account for Fe dimerisation. However, the number of available independent parameters, dictated by the Nyquist theorem 
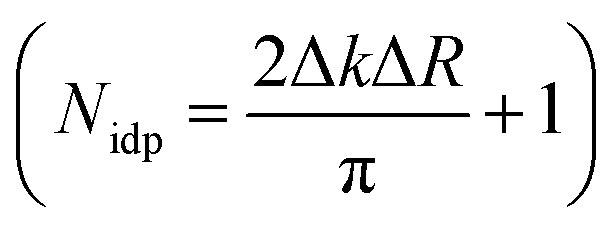
^41^ prohibits the inclusion of multiple Fe ions within the model.

### 3.2 X-ray absorption near-edge structure (XANES)

**Fig. 4:**
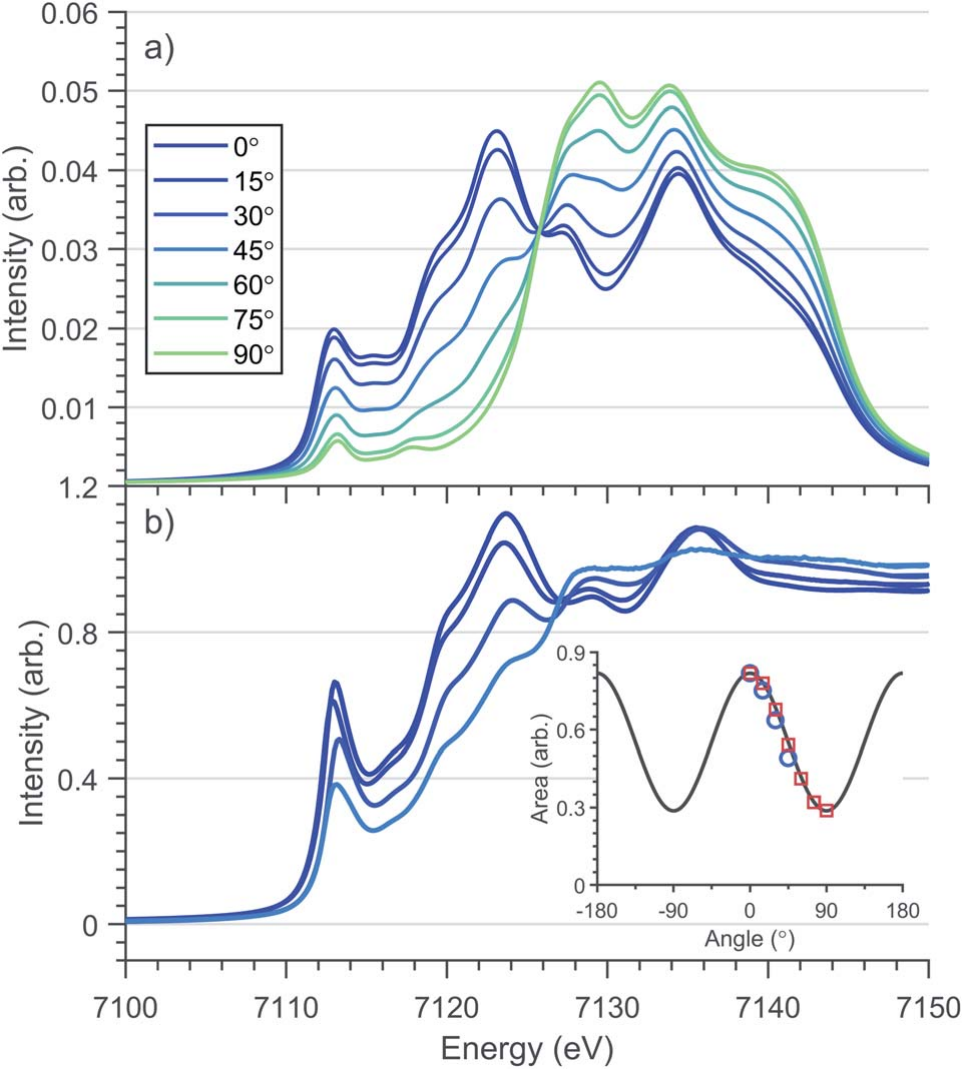
Single crystal angular dependent Fe K-edge XANES of Li_2_(Li_1−*x*_Fe_*x*_)N (a) DFT calculations (b) experimental spectra with a nominal concentration of *x* = 0.015. 0° corresponds with *E*⊥*c* and 90° with *E*∥*c*. (Inlay) Angular dependence of 1s–4p_*x*,*y*_ transition intensity of experimental (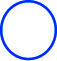) and theoretical (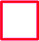) rising-edge peak centred at 7113 eV.

Transition metal oxidation state is frequently characterised by the K-edge threshold energy and the characteristic multiplet effects present within the K pre-edge.^42^ Fe K-edge XANES measurements on a polycrystalline sample of Li_2_(Li_1−*x*_Fe_*x*_)N was previously investigated by Niewa *et al.*^17,43^ The threshold energy region of the XANES was found to be dominated by an intense transition centred at 7113 eV. The origin of the 7113 eV peak was assigned by Niewa *et al.*^17,43^ as a K pre-edge (1s → 3d) transition, with enhanced intensity due to 4p-mixing, from which a local *C*_2v_ coordination symmetry was proposed. Since bending away from 180° introduces mixing of 4p_*x*_ character into 3d_*xz*_ and 4p_*y*_ into 3d_*yz*_ due to transformations under the same irreducible representations in *C*_2v_. To further investigate Fe site coordination symmetry, we preformed angular dependent single-crystal Fe K-edge XANES measurements of Li_2_(Li_0.985_Fe_0.015_)N, Fig. 4 and S2.† The area of the 7113 eV peak for each sample orientation gives the angular dependence of the transition oscillator strength, Fig. 4. Measurements were experimentally limited from 0–45°, where 0° corresponds with *E*⊥*c* and 90° with *E*∥*c*. Maximum intensity of the transition is observed at 0°. The variation in intensity as a function of crystal orientation follows a sinusoidal profile with a minimum at 90°; this is indicative of the two fold symmetry of dipole transitions with 4p_*x*,*y*_ orbital character. The XANES of linear Cu^I^ complexes are known to exhibit very low energy rising edge features due to transitions directly into degenerate 4p_*x*,*y*_ states,^44^ however the XANES of open-shell linear transition metal complexes are less developed. The 7113 eV feature in Li_2_(Li_0.985_Fe_0.015_)N is consistent with the crystal field model originally proposed for Cu^I^, whereby the linear coordination lifts the 4p_*x*,*y*,*z*_ degeneracy, decreasing the energy of the degenerate 4p_*x*,*y*_ orbitals that are non-bonding and increasing the 4p_*z*_ orbital to a higher energy. To obtain conclusion of the origin of the 7113 eV peak, and the associated local symmetry of the Fe site, DFT calculations based on a linear geometry were found to accurately reproduce the angular dependent XANES of Li_2_(Li_0.985_Fe_0.015_)N, Fig. 4. Projection of the density of states from this resultant DFT simulation verifies the interpreted splitting and degeneracy of the Fe-4p orbitals. Unoccupied character above the Fermi energy (Fig. S5†) coincides with the expected degeneracy of the 4p_*x*,*y*_ orbitals at the energy of the rising edge feature with 4p_*z*_ orbital character shifted to higher energy. In summary, our angular dependent K-edge XANES analysis on Li_2_(Li_0.985_Fe_0.015_)N identifies the local coordination symmetry involves a linear N–Fe–N motif; a conclusion that is further supported by our L_2,3_-edge analysis in the following section. The weak quadrupole allowed 1s → 3d K pre-edge transitions are however unresolved due to overlap with the considerably more intense 7113 eV feature. The absence of a resolvable pre-edge inhibits a ligand field multiplet analysis to quantitatively assign Fe spin ground-state by K-edge XANES.

### 3.3 Kβ X-ray emission spectroscopy

**Fig. 5:**
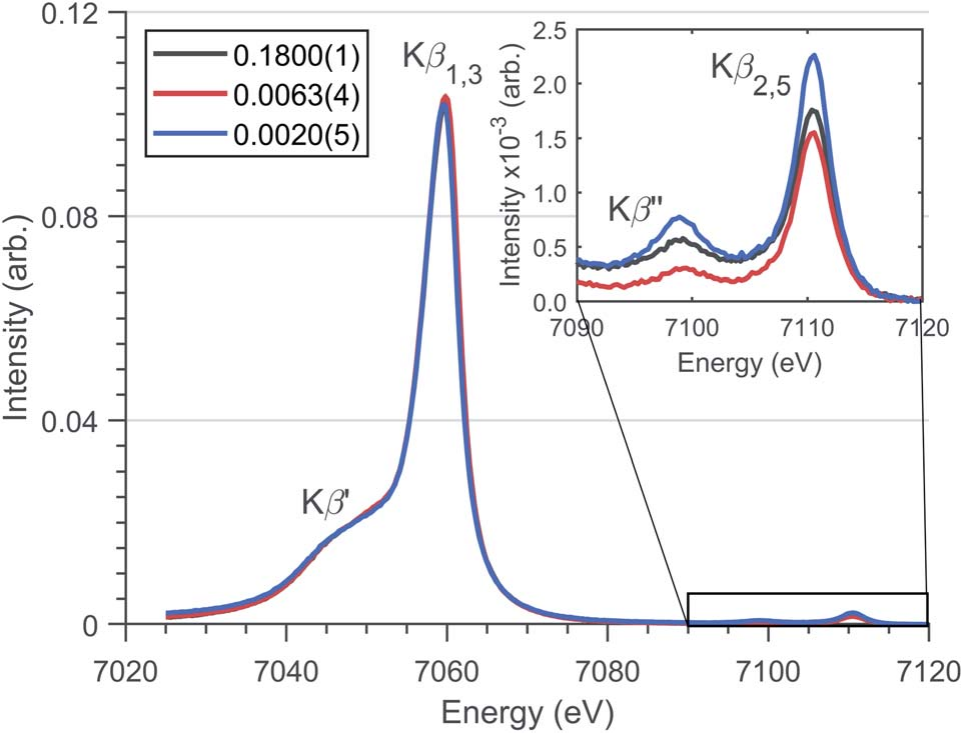
Fe Kβ XES spectrum of Li_2_(Li_1−*x*_Fe_*x*_)N for several concentrations (*x* values defined in legend) with spectral features highlighted. (Inlay) Magnified valence-to-core region.

Magnetisation measurements for Li_2_(Li_1−*x*_Fe_*x*_)N shows considerable dependence on *x.*^1^ In a recent theoretical study, many-body quantum chemistry calculations on [Fe^I^N_2_Li_14_]^9+^ and [Fe^II^N_2_Li_13_]^9+^ fragments show a strong reduction in magnetic anisotropy in going from a Fe^I^ to Fe^II^ dopant site.^20^ Previous studies have proposed that dopant sites couple to a finite number of Li ion vacancies giving rise to minority divalent species and a monovalent majority species with a ratio that changes as a function of dopant concentration.^20,39^ To address this question and investigate the spin-ground state of Li_2_(Li_1−*x*_Fe_*x*_)N, Kβ XES measurements were performed as a function of doping concentration, *x*, Fig. 5. Since Kβ utilises a non-resonant incident photon energy it is less affected by self-absorption than resonant techniques, making it ideally suited to studying the Fe concentration dependence of electronic structure. The Kβ mainline, Kβ_1,3_ and Kβ′, involves a dipole-allowed 3p–1s emission transition. These two mainline features have the advantage of being highly sensitive to spin ground-state, identified *via* differences in the Kβ_1,3_ and Kβ′ splitting and intensity.^10^Fig. 5 shows Kβ measurements for Li_2_(Li_1−*x*_Fe_*x*_)N where *x* = 0.1800(1), 0.0063(4) and 0.0020(5). There exists no electronic structural change as a function of doping concentration for Li_2_(Li_1−*x*_Fe_*x*_)N; confirming no valence change with respect to concentration. Frequently Kβ is applied to fingerprint the spin ground-state and to deduce the valence of transition metal ions. However, due to a lack of two coordinate Fe^I^ reference spectra, such qualitative analysis is inhibited. Despite this, Fig. S1† compares the Li_2_(Li_1−*x*_Fe_*x*_)N Kβ-mainline spectrum with a series of six coordinate *O*_h_ and *D*_4h_ Fe^II^ and Fe^III^ model complexes.^45^ The energy and intensity of the Kβ′′ first moment is consistent with either a d^6^ or d^7^ occupation of the Fe from which either *S* = 2 or 3/2 could be inferred. However, there is a significant deviation in the splitting and intensity between the Kβ_1,3_ and Kβ′ of Li_2_(Li_1−*x*_Fe_*x*_)N with respect to the model complexes.

The valence to core Kβ_2,5_ and Kβ′′ peaks have significant sensitivity to the local bonding environment around Fe site. The valence-to-core peaks at 7098.9 eV and 7110.6 eV (Fig. 5) correspond with metal character present within nitrogen 2s and 2p orbitals respectively. The lack of variation in the relative intensities and energies of these features is consistent with no variation in geometry around the N–Fe–N motif as a function of dopant concentration.

### 3.4 L_2,3_-edge X-ray absorption spectroscopy

**Fig. 6:**
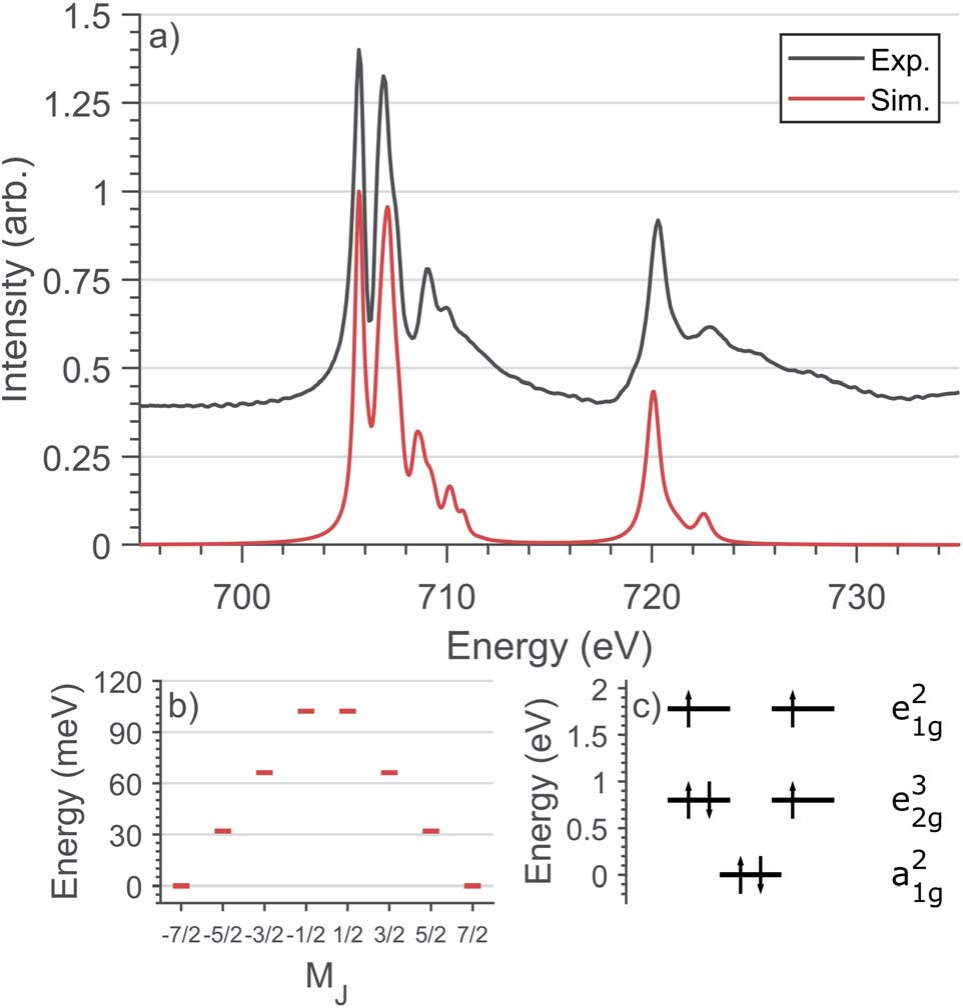
(a) Comparison of experimental and simulated Fe L_2,3_-edge spectra at 4.5 K with L_3_ peak normalisation. (b) The calculated *M*_J_ splitting of the ground-state *J* = 7/2 manifold. (c) The calculated energies of 3d orbitals obtained from the L_2,3_-edge ligand field multiplet fit.

L_2,3_-edge XAS accesses the electronic structure at the 3d orbitals through dipole allowed 2p–3d transitions. Single crystal measurements were performed with *E*⊥ to the crystallographic *c* axis and nominal doping concentration, *x* = 0.015. Fig. 6a shows the Fe L_2,3_-edge total-fluorescence spectrum measured at 4.5 K, with L_3_ and L_2_ edge peaks at 705.7 eV and 720.3 eV respectively. The L_3_-edge exhibits two intense features separated by 1.3 eV whereas the L_2_-edge is dominated by a single intense peak. Both L_2,3_-edges exhibit a series of high energy satellite features indicating the presence of significant metal–ligand charge transfer.

**Fig. 7:**
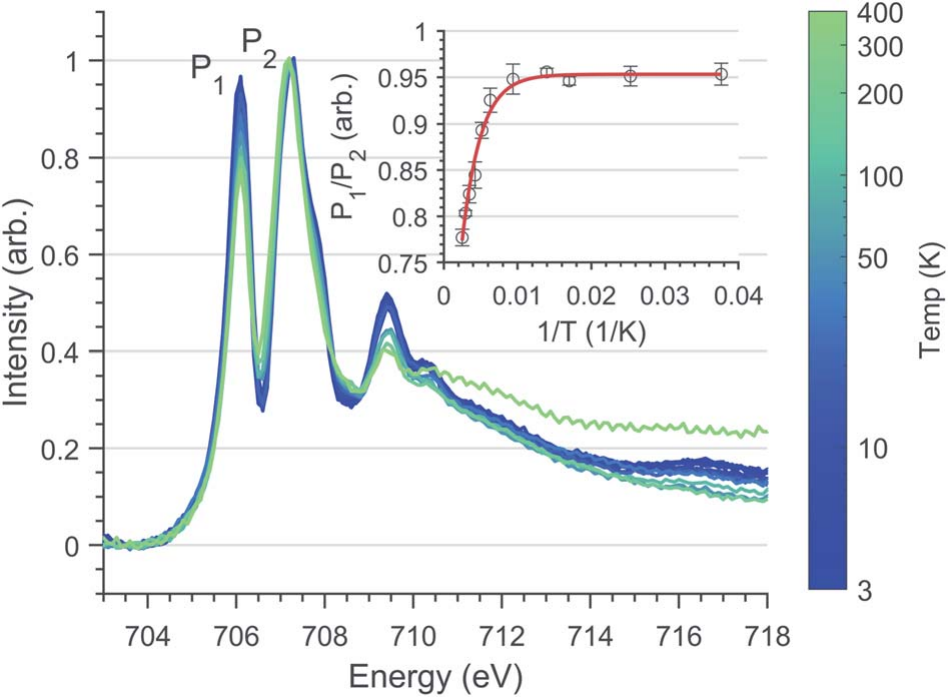
Fe L_3_ temperature dependence spectra of Li_2_(Li_0.98_Fe_0.02_)N normalised through *P*_2_ intensity. (Inlay) Least-squares fit of Eqn.(1) to L_3_ peak ratio with respect to the inverse absolute temperature. Error bars represent standard deviation of repeat measurements.

L_2,3_-edge XAS ligand field multiplet fitting was performed to quantify the 3d electronic structure of dopant sites. Two sets of simulations were performed based on both Fe^I^ and Fe^II^ scenarios. Initial fit parameters were extracted from the results of *ab initio* results reported by Xu *et al.*,^20^ see Table S1 and Fig. S6.† Optimisation of the simulated spectral features relative to experiment were obtained through adjustment of the ligand field parameters (*D*_t_ and *D*_s_) and include the effect of 4s mixing through the reduction in energy of the 3d_*z*^2^_ orbital. Agreement with the measured spectrum could only be obtained for the Fe^I^ valence model, with best fit parameters of *D*_q_ = 0, *D*_t_ = 0.1806 and *D*_s_ = −0.0257 eV corresponding to an electronic configuration such that d_*z*^2^_ has the lowest orbital energy, Fig. 6. The simulation quantifies the Li_2_(Li_0.985_Fe_0.015_)N dopant site as a Fe^I^ 3d^7^, ^4^*D*_7/2_ ion, with a ^5^*E* symmetry ground state resulting from a e_1g_^2^e_2g_^3^a_1g_^2^ configuration. The experimentally determined ligand field splitting is larger than the reported CASSCF result for a [Fe^I^N_2_Li_14_]^9+^ fragment that gave energies of 0, 0.91, 1.5 eV for a_1g_^2^, e_2g_^3^ and e_1g_^2^ respectively.^20^ The energy reduction in d_*z*^2^_ is approximately twice the value calculated for the linear monovalent SIM, [Fe(C(SiMe_3_)_3_)_2_]^−^.^6^ Spin–orbit coupling splits the total angular momentum of Fe sites into four Kramers doublets. In order of increasing energy these doublets, *M*_J_ = ±7/2, ±5/2, ±3/2 and ± 1/2, are evenly separated by approximately 2/3*ξ*. This splitting characterises the magnetic anisotropy barrier to the slow magnetic relaxation observed in Li_2_(Li_0.985_Fe_0.015_)N, Fig. 6b. At sufficiently low temperature only the *M*_J_ = ±7/2 Kramers doublet is populated. The 4.5 K L_2,3_-edge transitions hence emanate exclusively from the *M*_J_ = ±7/2 doublet. To evaluate the magnetic anisotropy barrier precisely, temperature dependent L_2,3_-XAS measurements were performed. We find the strong selection rules of L_2,3_-edge XAS makes the technique particularly sensitive to the population of *M*_J_ states. Therefore, *via* a series of measurements from 4.5 to 400 K the thermal population of *M*_J_ excited states can be experimentally deduced from changes in the line shape of the L_3_-edge spectrum. The temperature dependence is most clearly identified through the relative peak intensity for Peak 1 (*P*_1_), *E* = 706.1 eV *versus* Peak 2 (*P*_2_), *E* = 707.3 eV at the L_3_-edge, Fig. 7. Modelling the temperature dependent ratio of *P*_1_ and *P*_2_ through Maxwell–Boltzmann statistics the thermal population of the excited states can be achieved through the equation:1
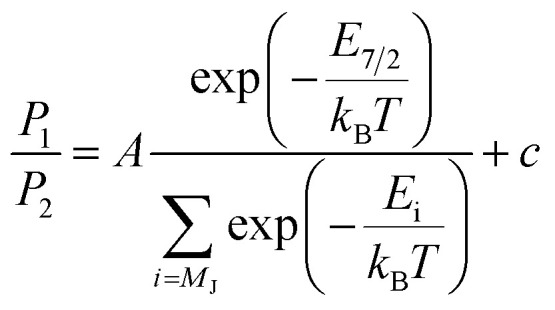
where *E*_*i*_ represents the energy of the four *M*_J_ ±7/2, ±5/2, ±3/2 and ±1/2 states (each evenly separated by 2/3*ξ*), *k*_B_ is the Boltzmann constant and *A* and *c* are multiplicative and scaling factors. The inlay of Fig. 7 shows L_3_-edge peak ratio (*P*_1_/*P*_2_) *versus* inverse absolute temperature (1/*T*) for Li_2_(Li_1−*x*_Fe_*x*_)N. Fitting to eqn (1) gives *ξ* = 52.2 ± 4.97 meV (421 ± 40.08 cm^−1^), significantly greater than the atomic value for Fe^I^ of 44.8 meV.^6^ Typically, the free ion spin–orbit coupling parameter represents the upper limit for spin–orbit coupling, where bonding leads to only decrease *ξ*. However, atomic spin–orbit coupling is strongly dependent on electron configuration, particularly on the number of 3d electrons.^46^ For instance, the 3d atomic spin–orbit coupling for a 3d^6^4s^1^ configuration is *ξ*_3d_ = 51 meV, approximately 6 meV greater than the value for a 3d^7^ configuration.^47^ Therefore, we propose that the measured value of *ξ* is greater than the 3d^7^ atomic value due to strong 4s–3d_*z*^2^_ mixing. Previously reported measurements of magnetic relaxation for Li_2_(Li_1−*x*_Fe_*x*_)N at low doping concentrations gave the effective energy barrier to magnetisation reversal (*U*_eff_) between 37.1 and 40.2 meV (298.9 and 324.6 cm^−1^).^12,19^ This is close to 34.80 ± 3.31 meV (280.7 cm^−1^) the energy splitting between the ground *M*_J_ = ±7/2 and first excited ±5/2 doublet determined from our variable temperature L_2,3_-edge XAS analysis. To our knowledge the application of variable temperature L_3_-edge XAS has not been previously reported. Therefore, we performed supporting calculations into the origin of this effect. This enabled us to confirm the same temperature dependence in the simulated spectra and test the validity of our fitting method (Fig. S7†). Furthermore, to identify the origin of the temperature dependence Fig. S8† shows the calculated L_2,3_-edge XAS spectra associated with each thermally populated Kramer's doublet of the ground-state *J* = 7/2 manifold. The calculations identify the individual intensity contributions to *P*_1_ and *P*_2_ for each *M*_J_ doublet.

**Fig. 8:**
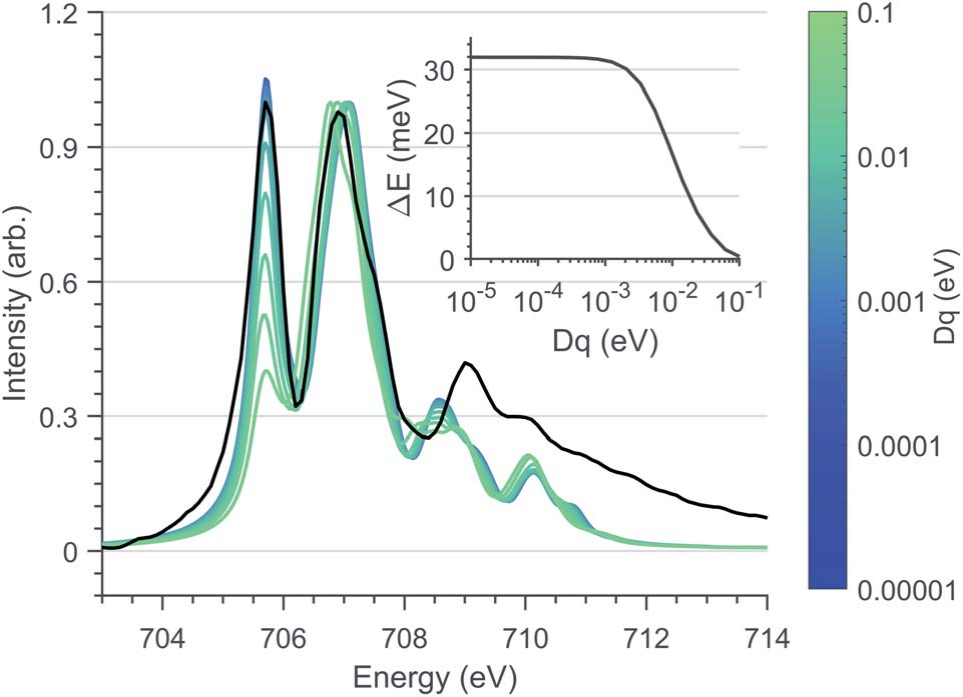
Ligand field multiplet calculations of the Fe L_3_-edge as a function of *D*_q_ splitting, normalised through second peak intensity. The *D*_t_ and *D*_s_ crystal field and spin–orbit coupling parameters are fixed to the optimised vales used in Fig. 6a. (Black) Experimental spectrum of Li_2_(Li_0.985_Fe_0.015_)N. (Inlay) Energy separation, Δ*E* of *M*_J_ ±7/2 and ±5/2 states with respect to *D*_q_.

Since first-order spin–orbit coupling in Li_2_(Li_1−*x*_Fe_*x*_)N is a manifestation of an odd electron count within the E_2g_ orbitals, the relationship between non-linearity due to N–Fe–N bending and the magnetic anisotropy barrier can be explored through the introduction of a *D*_q_ crystal field parameter. Fig. 8 shows the effect of including a non-zero *D*_q_ energy on the simulated Fe L_3_-edge. To maintain the measured anisotropy energy of Li_2_(Li_0.985_Fe_0.015_)N, the magnitude of *D*_q_ must be less than 1 meV. This result supports our angular dependent K-edge XANES analysis, demonstrating the strict N–Fe–N linearity imposed within the α-Li_3_N matrix.

## 4 Conclusion and outlook

We have characterised the local geometric and electronic structure of Fe dopant sites in Li_2_(Li_1−*x*_Fe_*x*_)N *via* K-edge XANES and EXAFS, L_2,3_-edge XAS and Kβ XES as a function of *x*, with particular attention to low values of *x*, where Fe sites are sufficiently isolated and hence perform as single ion magnets. The complementary use of element specific X-ray spectroscopy techniques unambiguously answers a multitude of questions that had limited quantitative understanding of this system. Kβ XES analysis rules out previous arguments^20^ for a divalent sub-species in Li_2_(Li_1−*x*_Fe_*x*_)N at low *x* concentration. EXAFS analysis shows no evidence of preferential Fe clustering at low dopant concentrations. L_2,3_-edge XAS measurements in conjunction with ligand field multiplet simulations conclude Fe sites are monovalent with a ^4^*D*_7/2_ ground state of ^4^*E* symmetry resultant from a a_1g_^2^e_2g_^3^e_1g_^2^ configuration. The energetic order of the 3d orbitals is affected by strong 4s–3d_*z*^2^_ mixing that results in a fully occupied 3d_*z*^2^_ at lowest energy. The strong influence of 4s–3d_*z*^2^_ mixing in reducing the destabilisation of 3d*σ* anti-bonding has long been reasoned by DFT and more recently by quantum chemistry calculations.^6,48^ It is shown that L_2,3_-edge XAS enables experimental quantification of 3d_*z*^2^_ energy reduction. The large 3d_*z*^2^_ energy reduction contributes to raising the anisotropy barrier in Li_2_(Li_1−*x*_Fe_*x*_)N, and is much greater than values calculated for other linear monovalent SIMs, including the first two coordinate complex, [Fe(C(SiMe_3_)_3_)_2_]^−^.^6^ Focusing then on low concentration Li_2_(Li_1−*x*_Fe_*x*_)N, variable temperature L_3_-edge XAS measurements from 4.5 to 400 K enabled quantification of the magnetic anisotropy barrier to slow magnetic relaxation. Analysis of variable temperature L_3_-edge XAS enables determination of the ground state *J* = 7/2 energy splittings. The splitting between the ground *M*_J_ = ±7/2 doublet and first excited *M*_J_ = ±5/2 doublet is 34.80 ± 3.31 meV (280.7 cm^−1^), this value is consistent with reported *U*_eff_ values obtained from magnetic relaxation measurements (37.1 and 40.2 meV (298.9 and 324.6 cm^−1^)^12,19^) identifying that magnetisation reversal manifests *via* this through barrier process. The *U*_eff_ is larger than reported values for other linear Fe SIMs, including [Fe(C(SiMe_3_)_3_)_2_]^−^ (*U*_eff_ = 22.4 meV, 181 cm^−1^),^6^ but is less than the value recently reported for a linear Co complex Co^II^(C(SiMe_2_ONaph)_3_)_2_ (55.8 meV, 450 cm^−1^)^4^ and lanthanide SIMs which can exceed 150 meV.^49^ Despite the unexceptional *U*_eff_ energy for Li_2_(Li_1−*x*_Fe_*x*_)N, the relaxation time at low temperatures certainly is exceptional *τ* = ∼10^7^ s (ref. 19) in comparison with other linear SIMs where *τ* is within a range of seconds and less.

To further understand the origin of the unusually long Li_2_(Li_1−*x*_Fe_*x*_)N relaxation time, we have analysed the geometric structure and coordination symmetry around Fe dopant sites. EXAFS analysis find both Fe–N bond lengths as 1.873(7) Å, which is exceptionally short for two-coordinate Fe^I^. The shortness of the Fe–N bonds suggests strong Fe–N π bonding, facilitated by the *D*_6h_ point symmetry providing equal N 2p π-mixing into both 3d_*xz*_ and 3d_*yz*_ orbitals.^50^ Further evidence of this is observed *via* strong satellite intensities present in the L_2,3_-edge XAS spectra. The N–Fe–N angle is analysed by K-edge XANES through single-crystal angular dependence of an intense, low energy, 7113 eV peak. A ligand field interpretation is backed up by DFT calculations, assigning the transition as being associated with unoccupied 4p_*x*,*y*_ orbitals, from which it is deduced that the N–Fe–N bonding does not deviate from linear. This conclusion is supported by ligand field multiplet simulations that indicate that *D*_q_ induced degeneracy breaking of 3d_*xy*_ and 3d_*x*^2^−*y*^2^_ cannot exceed 1 meV for the measured energy reversal barrier to be maintained.

Together the X-ray spectroscopy results identify Li_2_(Li_1−*x*_Fe_*x*_)N as an ideal model system clean of stoichiometric vacancies where Fe sites are geometrically equivalent. The doping of Fe ions into the lithium nitride host matrix enables control of inter-SIM distances, from which dipolar fields can be minimised. The introduction of Fe sites displace Li ions at 2*c* positions causing a local bond contraction of 0.062(7) Å with respect to the equivalent Li–N bond. The linear N–Fe–N core is supported through 3d_*xz*,*yz*_-Nπ mixing and indirectly by the hexagonal lithium nitride lattice, that acts to drive bond shortening and rigorous linearity, in a similar but more direct way than dispersion force stabilisation observed in other linear molecular complexes, including Fe[N(SiMe_3_)Dipp]_2_.^38^

Previous theoretical studies have identified the crucial influence of reduced symmetry and Renner–Teller vibronic coupling on the magnetic relaxation time in two coordinate Fe SIMs.^51^ It is proposed that the combination of a short Fe–N bond, related strong 3dπ bonding, and high point symmetry imposed by the hexagonal lithium nitride lattice contribute to suppress vibronic effects, resulting in increased magnetic relaxation times with respect to other linear SIMs. The high point symmetry of the solid-state host lattice exhibit less disorder with respect to large inorganic coordination complexes. The high symmetry of the crystal host lattice and geometric equivalence of Fe dopant sites, result in a very low propensity for dislocation-induced strain type variations in local symmetry and easy axis directions, consistent with the extreme field dependence reported in Li_2_(Li_1−*x*_Fe_*x*_)N.^13,19^

The quantification of electronic structure reported here provides insights relevant for the advance of high performance magnets free from rare-earth metals. The extraordinary electronic and magnetic properties of Li_2_(Li_1−*x*_Fe_*x*_)N, highlights the potential of doping paramagnetic ions within high symmetry solid-state lattices. Another area of potential relevance is nano-scale information storage for which there is currently considerable effort devoted to depositing coordination complexes with SIM properties on surfaces.^52,53^ An even distribution of SIM dopant sites within a high symmetry host lattice crystal or thin film^54^ offers an interesting alternative method, with additional degrees of freedom for controlling local symmetry and lattice phonon dispersion.

## Conflicts of interest

There are no conflicts to declare.

## Supplementary Material

SC-011-D0SC03787G-s001
