# Supplementary material for: Single-ion magnetism in the extended solid-state: insights from X-ray absorption and emission spectroscopy
Source: Chem Sci. 2020 Oct 7;11(43):11801–10. doi: 10.1039/d0sc03787g (PMC8162461; doi:10.1039/d0sc03787g)
Supplement: SC-011-D0SC03787G-s001 [file SC-011-D0SC03787G-s001.pdf]

Electronic Supplementary Material (ESI) for Chemical Science.
This journal is © The Royal Society of Chemistry 2020

Electronic Supplementary Information:

Single-Ion Magnetism in the Extended Solid-State: Insights from X-ray Absorption and Emission Spectroscopy

Myron S. Huzan,[a b] Manuel Fix,[c] Matteo Aramini,[d] Peter Bencok,[d] J. Fred Mosselmans,[d] Shusaku Hayama,[d] Franziska A. Breitner,[c] Leland B. Gee,[e] Charles J. Titus,[e] Marie-Ann Arrio,[f] Anton Jesche,[c] Michael L. Baker[a b]

[a] *The University of Manchester at Harwell, Diamond Light Source, Harwell Campus, OX11 0DE, UK*

[b] *Department of Chemistry, The University of Manchester, Manchester, M13 9PL, UK*

[c] *EP VI, Center for Electronic Correlations and Magnetism, Institute of Physics, University of Augsburg, D-86159 Augsburg, Germany*

[d] *Diamond Light Source, Harwell Science and Innovation Campus, Chilton, Didcot, OX11 0DE, UK*

[e] *Department of Chemistry, Stanford University, Stanford, CA 94305, USA*

[f] *Institut de Minéralogie, de Physique des Matériaux et de Cosmochimie, CNRS, Sorbonne Université, IRD, MNHN, UMR7590, 75252 Paris Cedex 05, France.*

Contents

S.1 Kβ Comparison . . . 3
S.2 Experimental XANES Results . . . 4
S.3 $L_{2,3}$-edge background subtraction . . . 5
S.4 Density of States . . . 6
S.5 $L_{2,3}$-edge Experimental vs CASSCF . . . 7
S.6 Quanty Calculation of Anisotropy Barrier . . . 9

S.1 Kβ Comparison

To the authors knowledge there have been no published data of Kβ measurements performed on transition metal ions in linear complexes. Comprehensive studies are documented for Fe^{II} and Fe^{III} of standard symmetries (O_h and D_{4h}), Figure S1 which provides the only comparison for the measured spectrum. Intensity was normalised to the integrated trapezoidal area of each spectra. $Li_2(Li_{1-x}Fe_x)N$ displays closest resemblance to the $S = 2$ or 3/2 Fe model complexes which infer a d^6 or d^7 occupation respectively. However, there exists a strong deviation of K$\beta_{1,3}$ and Kβ' energy splitting and relative intensity limiting further comparative deductions to model complexes.

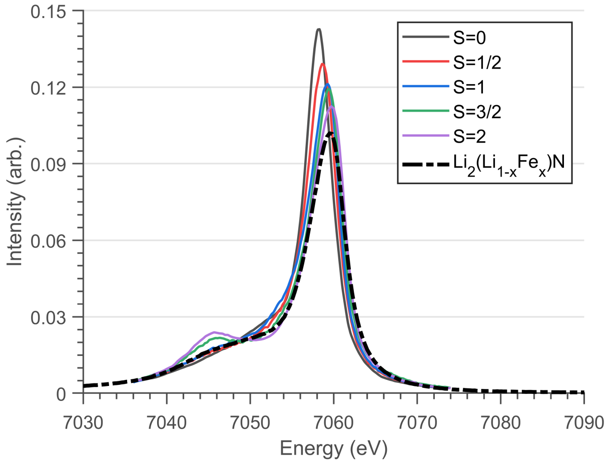

Figure S1: Kβ fluorescence mainline comparison of $Li_2(Li_{1-x}Fe_x)N$ with model Fe^{III} and Fe^{II} complexes with various spin moments of standard symmetries (O_h and D_{4h})[1].

S.2 Experimental XANES Results

XANES displays minimal spectral differences as a function of doping concentration, Figure S2. Slight discrepancies are likely correlated to fluctuating background subtraction or self-absorption effects. Rising edge feature (7113 eV) was isolated and fit with a two peak Pearson-VII fitting routine to extract transition angular dependence described by total area of the peak.

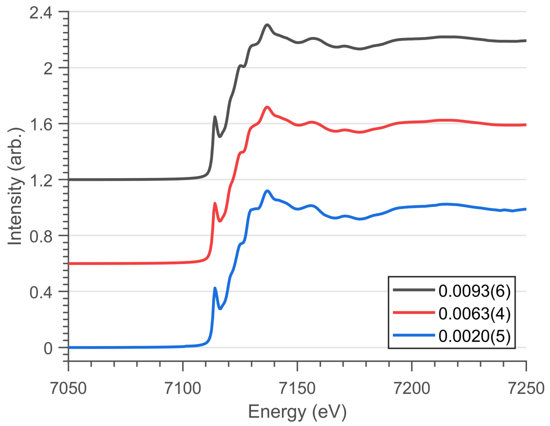

Figure S2: Fe K-edge XANES spectra for various concentrations, x (Values defined in legend) of $Li_2(Li_{1-x}Fe_x)N$.

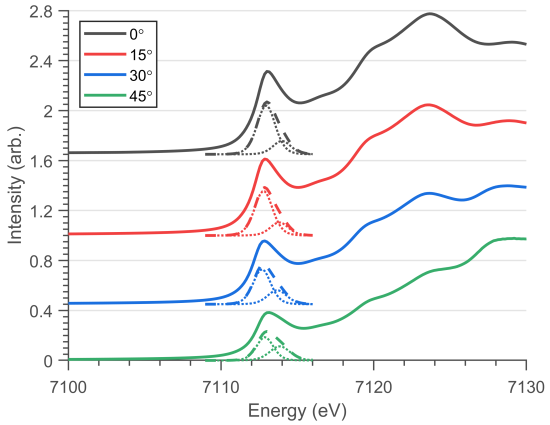

Figure S3: Angular dependent Fe K-edge XANES fitting, isolating the rising-edge peak of $Li_2(Li_{0.985}Fe_{0.015})N$. Dotted lines represent fitted Pearson-VII peaks and dashed lines the total fitted rising-edge area.

S.3 $L_{2,3}$-edge background subtraction

Figure S4 illustrates the background normalisation steps undertaken for the Fe-$L_{2,3}$ spectra. Pre- and Post-Edge normalisation is achieved through a linear fit, while Equation S1 models the background subtraction for the $2p_{3/2}$ and $2p_{1/2}$ continuum states through a double arc-tangent function [2].

$$f(2p_{ctm}) = \frac{h_{L3}}{\pi}(\arctan(k \cdot (E - E_{L_3})) + \frac{h_{L2}}{\pi}(\arctan(k \cdot (E - E_{L_2})) \tag{S1}$$

Heights of the arc-tangent (h_{L_3} and h_{L_2}) were set to the approximate minima of each edge while the energy shift is defined as the inflection point of the corresponding edges, $E_{L_3} = 705.7$ eV and $E_{L_2} = 720.3$ eV, $k = 2.0$.

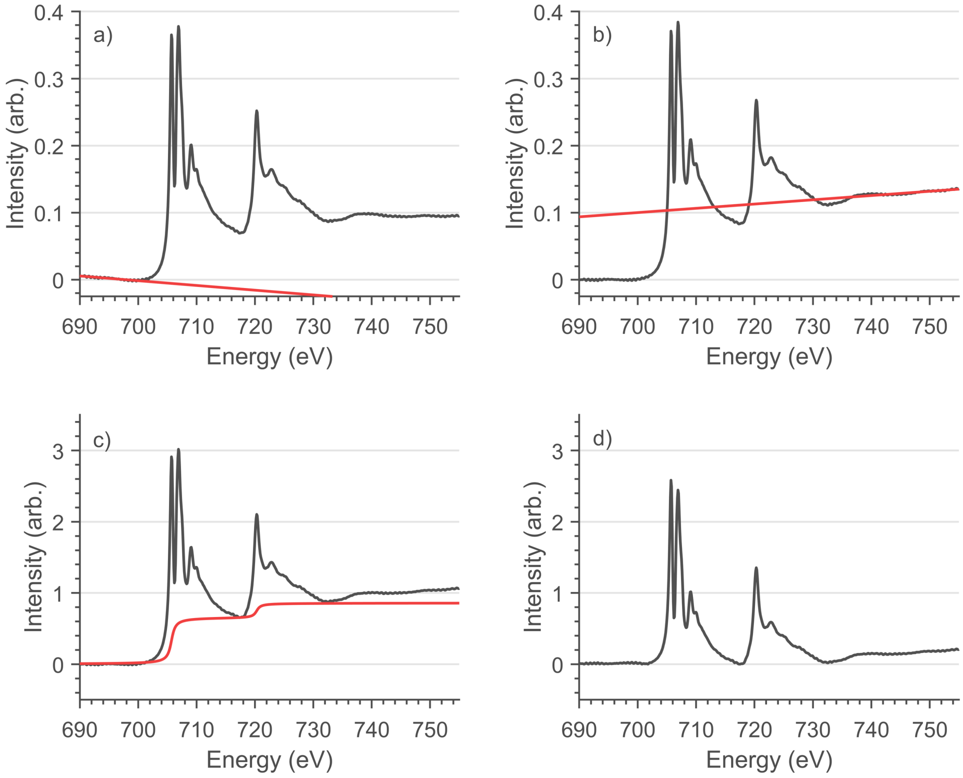

Figure S4: a) Background subtraction through linear fitting to pre-edge (690 - 700 eV), b) Normalisation of spectra through linear fitting to post-edge (735 - 750 eV), c) 2p continuum subtraction of equation S1, d) Normalised and background subtracted $L_{2,3}$-edge spectra of $Li_2(Li_{1-x}Fe_x)N$.

S.4 Density of States

As described in the main body of text DFT calculations were performed within Quantum-Espresso[3] and CASTEP[4] of a 3x3x3 supercell Li_3N matrix doped with a single Fe atom at the Wyckoff 1***b*** position. Interpretation of the converged system included mapping the partial density of states (pDOS), Figure S5. pDOS calculations are an extension of Mulliken population analysis capable of isolating the individual bands and orbitals of a selected atom. Unoccupied $4p_{x,y}$ states characterise the observed rising-edge feature, 7113 eV and $4p_z$ character is shifted to higher energy, as predicted for a linear coordination through a crystal field model.

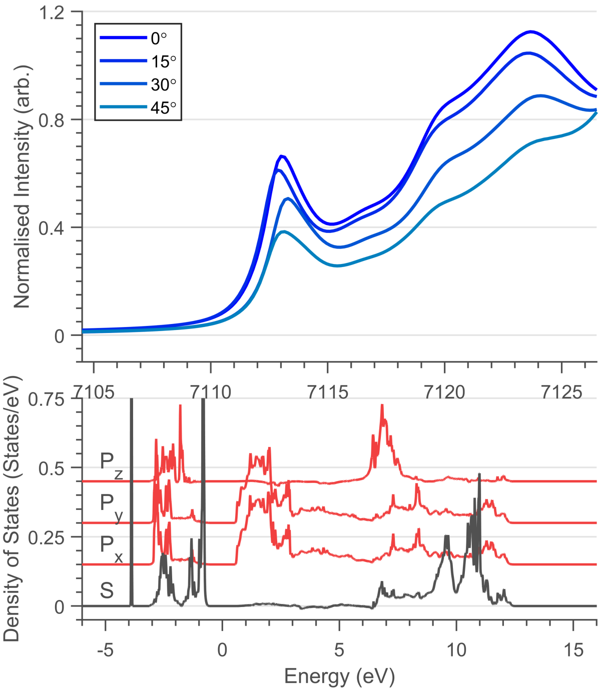

Figure S5: (*Top*) Experimental K-edge XANES of $Li_2(Li_{1-x}Fe_x)N$ with nominal concentration x = 0.0015. (*Bottom*) LDA+U Partial Density of States analysis of Fe s and p states

S.5 $L_{2,3}$-edge Experimental vs CASSCF

ab initio CASSCF calculations by Xu *et al.* predict the experimentally measured magnetic anisotropy of $Li_2(Li_{1-x}Fe_x)N$ to be ∼27 meV with either a linear Fe^{I} or quasilinear Fe^{II} coordination complexes[5]. Figure S6 shows Quanty[6] simulations of $L_{2,3}$-edge spectra of Fe^{I} and Fe^{II} valences. The Slater-Condon-Shortley parameters are reduced to 80% of the calculated Hartree-Fock values to account for over-estimation of the free ion values. Crystal field splittings were extracted from CASSCF calculations, Table S1. These simulations indicated Fe(I) provided the most suitable fit from which crystal field splitting was optimised to best reproduce the experimentally measured spectra.

Table S1: Quanty[6] fitting parameters of simulations presented in Figure 6. Dq, Dt and Ds represent the ligand field splitting parameters of a $D_{6\ h}$ symmetry and ξ_{3d} the spin-orbit coupling parameter deduced from Hartree-Fock calculations. Dq set to zero for all calculations. All values defined in eV.

		CASSCF[5]	
	Best Sim.	Fe(II)	Fe(I)
Dt	0.1806	0.1480	0.1829
Ds	-0.0257	-0.2300	-0.0786
ξ_{3d}	0.0522	0.039	0.045

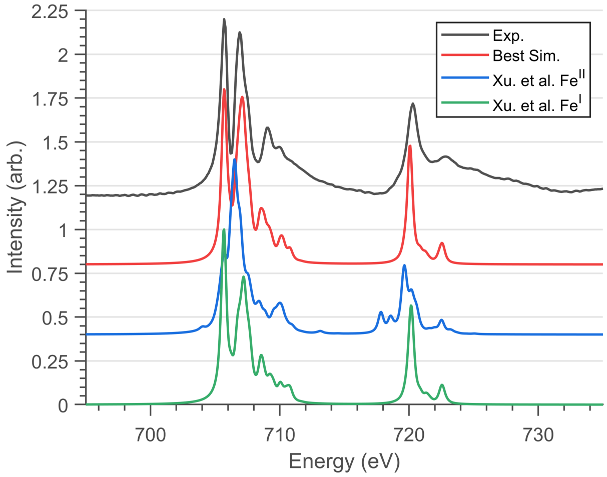

Figure S6: Comparisons of experimental (black), best simulated (red) and Xu *et al.*[5] Fe(II) (blue) and Fe(I) (green) models of Fe $L_{2,3}$-edge spectra. Calculation and experimental measurements undertaken at 4.5 K with L_3 peak maximum normalisation (704 - 708 eV). Ligand field parameters defined in Table S1.

S.6 Quanty Calculation of Anisotropy Barrier

Quanty calculations achieve a second quantisation approach of analytically simulating core-level spectroscopy[6]. Through this technique expectation values of quantum mechanical operators can be applied to the wavefunctions designated to the n electrons present within the basis set. As a result of this energy level separation each M_J doublet can be assigned and quantified.

To replicate the magnetic anisotropy determination undertaken experimentally, a temperature dependent L_3-edge simulation was performed, Figure S7. Fitting of Equation 1 determines an anisotropy energy of $E = 36.87 \pm 0.22$ meV between the ground $M_J = \pm 7/2$ and first excited state, $\pm 5/2$. As reported for the experimental determination of the anisotropy barrier, the calculation assumes equal splitting of the M_J ground-states.

This result is aligned with all previously experimentally and theoretically deduced values of magnetic anisotropy[7,8] and corroborates the technique of utilising $L_{2,3}$-edge temperature dependence to extract magnetic anisotropy of complex systems.

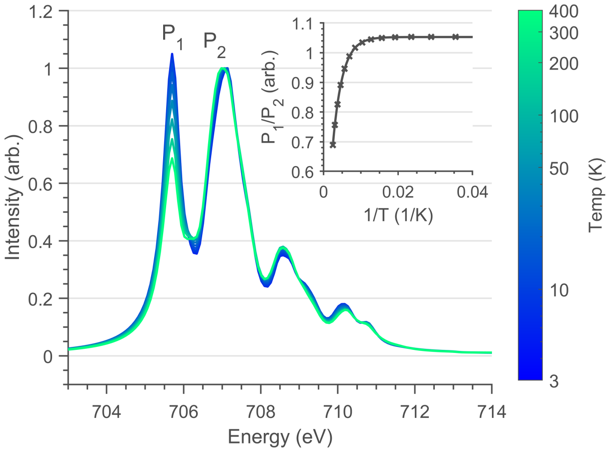

Figure S7: Quanty calculations of Fe L_3-edge as a function of temperature. Normalised through P_1 intensity. (*Inlay*) Least-squares fit of Equation 1 to Quanty simulation L_3 peak ratio with respect to the inverse absolute temperature.

Figure S8 isolates the spectral contribution of each populated M_J doublet to identify the spectral dependence relative to the predominant P_1 and P_2 features. This figure shows that the intensity of P_1 is uniquely related to the $\pm 7/2$ state, verifying the relationship between the temperature

dependence of the P_1 to P_2 ratio and the magnetic anisotropy energy.

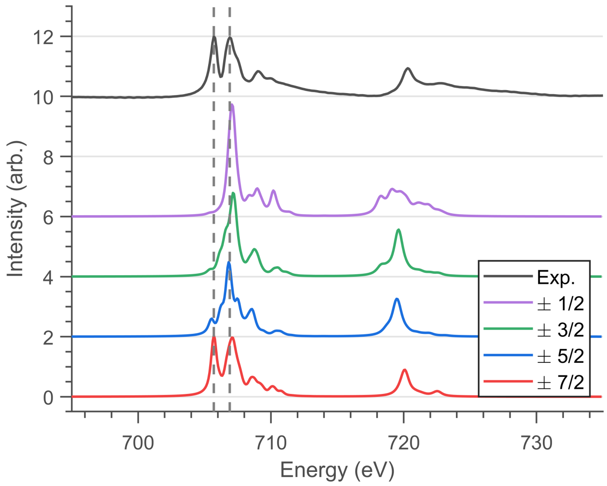

Figure S8: Fe $L_{2,3}$ Quanty calculations of the individual M_J ground-states compared to the experimental $Li_2(Li_{1-x}Fe_x)N$ spectra. Vertical dashed lines represent P_1 and P_2.

Table S2: Expectation values for the J=7/2 manifold based on parameters obtained from the best fit to the $L_{2,3}$-edge spectrum of Fe^{I}, corresponding to the d-orbital electron occupation, spin, S, orbital angular momentum, L and total angular momentum, J, oriented parallel to the principle axis, corresponding with the crystallographic c axis.

E (meV)	$\langle d_{xy} \rangle$	$\langle d_{xz} \rangle$	$\langle d_{z^2} \rangle$	$\langle d_{yz} \rangle$	$\langle d_{x^2-y^2} \rangle$	$\langle S_z \rangle$	$\langle L_z \rangle$	$\langle J_z \rangle$
0	0.9998	1.0002	1.9995	1.0005	2.0000	1.4992	2.0008	3.5000
0	2.0000	1.0005	1.9995	1.0002	0.9998	−1.4992	−2.0008	−3.5000
31.94	1.9885	1.0010	1.9991	1.0005	1.0109	−0.4893	−1.9555	−2.4448
31.94	1.0109	1.0005	1.9991	1.0010	1.9885	0.4893	1.9555	2.4448
66.17	1.9993	1.0010	1.9991	1.0003	1.0003	0.4987	1.9987	1.5000
66.17	1.0003	1.0003	1.9991	1.0010	1.9993	−0.4987	−1.9987	−1.5000
102.19	1.9994	1.0006	1.9994	0.9996	1.0010	−1.4978	−1.9978	−0.5000
102.19	1.0010	0.9996	1.9994	1.0006	1.9994	1.4978	1.9978	0.5000

References

[1] W. Zhang, R. Alonso-Mori, U. Bergmann, C. Bressler, M. Chollet, A. Galler, W. Gawelda, R. G. Hadt, R. W. Hartsock, T. Kroll, K. S. Kjær, K. Kubiek, H. T. Lemke, H. W. Liang, D. A. Meyer, M. M. Nielsen, C. Purser, J. S. Robinson, E. I. Solomon, Z. Sun, D. Sokaras, T. B. Van Driel, G. Vankó, T. C. Weng, D. Zhu and K. J. Gaffney, *Nature*, 2014, **509**, 345–348.

[2] E. C. Wasinger, F. M. De Groot, B. Hedman, K. O. Hodgson and E. I. Solomon, *Journal of the American Chemical Society*, 2003, **125**, 12894–12906.

[3] P. Giannozzi, S. Baroni, N. Bonini, M. Calandra, R. Car, C. Cavazzoni, D. Ceresoli, G. L. Chiarotti, M. Cococcioni, I. Dabo, A. Dal Corso, S. De Gironcoli, S. Fabris, G. Fratesi, R. Gebauer, U. Gerstmann, C. Gougoussis, A. Kokalj, M. Lazzeri, L. Martin-Samos, N. Marzari, F. Mauri, R. Mazzarello, S. Paolini, A. Pasquarello, L. Paulatto, C. Sbraccia, S. Scandolo, G. Sclauzero, A. P. Seitsonen, A. Smogunov, P. Umari and R. M. Wentzcovitch, *Journal of Physics Condensed Matter*, 2009, **21**, 395502.

[4] S. J. Clark, M. D. Segall, C. J. Pickard, P. J. Hasnip, M. I. Probert, K. Refson and M. C. Payne, *Zeitschrift fur Kristallographie*, 2005, **220**, 567–570.

[5] L. Xu, Z. Zangeneh, R. Yadav, S. Avdoshenko, J. Van Den Brink, A. Jesche and L. Hozoi, *Nanoscale*, 2017, **9**, 10596–10600.

[6] M. W. Haverkort, M. Zwierzycki and O. K. Andersen, *Physical Review B - Condensed Matter and Materials Physics*, 2012, **85**, 165113.

[7] A. Jesche, R. W. McCallum, S. Thimmaiah, J. L. Jacobs, V. Taufour, A. Kreyssig, R. S. Houk, S. L. Bud'Ko and P. C. Canfield, *Nature Communications*, 2014, **5**, 3333.

[8] M. Fix, J. H. Atkinson, P. C. Canfield, E. Del Barco and A. Jesche, *Physical Review Letters*, 2018, **120**, 147202.
